# Blood pressure and heart rate variability to assess autonomic response to an acute bout of high intensity interval exercise in healthy young adults

**DOI:** 10.14814/phy2.16142

**Published:** 2024-07-25

**Authors:** Saniya Waghmare, Alicen A. Whitaker‐Hilbig, Mark Chertoff, Sandra A. Billinger

**Affiliations:** ^1^ Department of Physical Therapy, Rehabilitation Science, and Athletic Training University of Kansas Medical Center Kansas City Kansas USA; ^2^ Department of Neurology University of Kansas Medical Center Kansas City Kansas USA; ^3^ Department of Physical Medicine and Rehabilitation Medical College of Wisconsin Milwaukee Wisconsin USA; ^4^ Cardiovascular Center Medical College of Wisconsin Milwaukee Wisconsin USA; ^5^ Department of Hearing and Speech University of Kansas Medical Center Kansas City Kansas USA; ^6^ Department of Physical Medicine and Rehabilitation University of Kansas Medical Center Kansas City Kansas USA; ^7^ University of Kansas Alzheimer's Disease Research Center Fairway Kansas USA; ^8^ Department of Cell Biology and Physiology University of Kansas Medical Center Kansas City Kansas USA

**Keywords:** autonomic nervous system, cardiovascular, coefficient of variation, physiology, spectral analysis

## Abstract

Autonomic nervous system (ANS) activity causes acute variations in blood pressure (BP) and heart rate (HR). These systems are challenged during high intensity interval exercise (HIIE). However, BP variability (BPV) and HR variability (HRV) response to HIIE is unknown. We characterized BPV and HRV during an acute HIIE bout using spectral low frequency [LF] and high frequency [HF] domains. We hypothesized that BPV would increase and HRV would decrease during high‐intensity and active‐recovery of HIIE compared to baseline [BL] and BPV would reduce and HRV would increase during cool down, post‐HIIE, and 30 min post‐HIIE compared to BL. HIIE involved 10 min of alternating high‐intensity and active‐recovery (approximately 70% and 10% of Wattmax) on a recumbent stepper. We did a secondary analysis on 23 datasets. The participants were 25 ± 1.5 years, 48% females. Our results showed high‐intensity BPV LF was not significantly different from BL while HF increased. HRV LF and HF decreased compared to BL. During active‐recovery, LF and HF for BPV and HRV increased greater than high‐intensity. HRV LF and HF returned to BL after 30 min of recovery, whereas BPV HF was higher compared to BL. The rapid switching during HIIE uniquely modulates cardiovascular and ANS.

## 1 INTRODUCTION

High intensity interval exercise (HIIE) includes alternating high intensity and recovery bouts (Gibala et al., 2012; MacInnis & Gibala, 2017). The autonomic nervous system (ANS) regulates key involuntary physiologic processes such as heart rate, blood pressure, and breathing at rest and during physiologic challenges such as exercise. While blood pressure and heart rate have shown to change concomitantly with exercise intensity during low‐volume HIIE in young healthy adults, (Whitaker et al., 2022) blood pressure or heart rate alone does not provide sufficient evidence about ANS modulation and cardiovascular hemodynamics.

The ANS is assessed both in research and clinically using heart rate variability (HRV) and beat‐to‐beat blood pressure variability (BTB BPV). Our current understanding during continuous incremental exercise is that BTB BPV increases due to neuro‐humoral factors such as sympathetic activity, vasodilators, metabolites, endothelial shear stress, total peripheral resistance and cardiac output influencing blood pressure to constantly maintain perfusion to muscles and vital organs (Bakkar et al., 2021; Cottin et al., 2008; Schutte et al., 2022). Whereas HRV reduces during higher intensity exercises, when heart rate reaches 120–180 beats/min due to enhanced heart rate that augments cardiac output and blood flow (Michael et al., 2017). Limited information is available regarding the ANS response during interval exercise. Therefore, characterizing BTB BPV and HRV during short‐interval, low‐volume HIIE in young healthy adults would provide information about the ANS and its influence on the cardiovascular system during changes in exercise intensity. Specifically, short‐interval HIIE includes intensity bursts of 30 to 60 s interspersed with active recovery, which may challenge the ANS in maintaining constant cerebral perfusion (Boyne et al., 2015). Based on the existing literature for continuous exercise, a working hypothesis may be that during the high‐intensity bout of HIIE, there would be an increase in BTB BPV and reduced HRV. During the recovery bouts of HIIE, decreases in exercise intensity and sympathetic activity may therefore reduce BTB BPV and increase HRV. However, BTB BPV and HRV may not return to resting levels, during the active recovery bouts and cooldown due to the continued exercise at low intensity, which would maintain increased sympathetic activity.

During the recovery cool down when both exercise intensity and cadence are decreasing back to a resting state, one would anticipate a withdrawal of sympathetic activity and an increase in parasympathetic activity that would increase HRV. However blood pressure and BTB BPV would decrease during recovery following HIIE. To reduce the occurrence of post‐exercise hypotension and syncope, the ANS and sympathetic activity may quickly return back to the resting values following exercise (Halliwill, 2001; Murrell et al., 2007). Therefore, understanding ANS through BTB BPV and HRV during changing intensities of HIIE and recovery in healthy young adults would provide foundational knowledge about the cardiovascular system responses and provide a framework for future comparison to people living with cardiovascular or neurological disease.

The primary aim of this investigation was to examine changes in BTB BPV during and following a single bout of short‐interval, low‐volume HIIE in young, healthy adults. Our secondary aim was to examine HRV during and following a single bout of HIIE. We hypothesized that for BTB BPV parameters quantified by the co‐efficient of variation (CoV), low frequency (LF) power spectral density (PSD), high frequency (HF) PSD, would increase during high‐intensity phase and active recovery phase of HIIE compared to BL. Whereas BTB BPV CoV, LF PSD, HF PSD would reduce during cool down after HIIE, immediately following HIIE, and 30 min after HIIE. We also hypothesized that HRV parameters, LF PSD, HF PSD would reduce during both high‐intensity phase and active recovery phase of HIIE compared to BL. Whereas HRV LF PSD, HF PSD would increase during cool down after HIIE, immediately following HIIE, and 30 min after HIIE.

## 2 METHODS

We performed a secondary analysis of existing data where the primary focus was cerebrovascular response to HIIE (Whitaker et al., 2022). Individuals enrolled into the study provided written informed consent prior to the study visits and were: (1) age 18–30 years old and (2) low cardiac risk defined by the American College of Sports Medicine (ACSM) (Thompson et al., 2013). For those included in this secondary analysis, participant characteristics are described in Table 1. The primary study was approved by University of Kansas Medical Center Human Subjects Committee.

**TABLE 1:** Participant characteristics.

	All	Women	Men	*p*‐value
*n*	23	12	11	
Age (years) ^∞^	25 (SD 1.65)	24.6 (SD 1.9)	24.9 (SD 1.0)	0.65
BMI, Kg/m^2∞^	23.6 (SD 3.6)	21.0 (3.26)	26.0 (1.89)	<0.001*
Race/ethnicity^ₒ^, *n* (%)				
White/Caucasian	17 (73.9%)	9 (75.0%)	8 (72.7%)	1.00
Hispanic/Latino	1 (4.3%)	0	1 (9.1%)	1.00
Asian	5 (21.7%)	1 (10%)	2 (18.2%)	1.00
Native American	1 (4.3%)	0	1 (9.1%)	1.00
Physical Activity score^ₒ^, *n* (%)				
<10 min, 5 time/week	1 (4.3%)	1 (8.3%)	0	
20–60 min/week	2 (8.7%)	1 (8.3%)	1 (9.1%)	
1–3 h/week	2 (8.7%)	1 (8.3%)	1 (9.1%)	
>3 h/week	18 (78.3%)	9 (75%)	9 (81.8%)	1.00
Estimated VO_2max_^∞^, mL.kg^−1^.min^−1^	44.6 (SD 6.4)	41.9 (SD 4.5)	47.1 (SD 7.1)	0.06

*Note*: Means (standard deviation). ^ₒ^Analyzed by Fisher exact test. ∞Analyzed by one way ANOVA.

Abbreviations: h/week, hours per week; kg/m^2^, kilogram of body weight per meter square; min/week, minute per week; mL.kg^−1^.min^−1^, milliliters of oxygen per kilogram of body weight per minute; VO_2max_, maximum oxygen consumption; BMI, body mass index.

* *p* < 0.05.

The methodology for the primary paper is presented in greater detail elsewhere (Whitaker et al., 2022). Briefly, participants performed a submaximal exercise test using a recumbent stepper (Billinger et al., 2012; Whitaker et al., 2023; Wilson et al., 2017) to determine the workload for the HIIE bout during their visit 1 (Boyne et al., 2023; Whitaker et al., 2022). Following a 20‐min rest on the recumbent stepper, participants were instrumented with equipment. For purposes of this study, we present the following variables of interest: (1) 5‐lead electrocardiogram (ECG; Cardiocard, Nasiff Associates, Central Square, NY) for heart rate, and (2) beat‐to‐beat blood pressure finger cuff (Finometer, Finapres Medical System, Amsterdam, The Netherlands) for mean arterial pressure (MAP).

Data was continuously recorded for 5 min of seated rest at baseline (BL). The HIIE protocol started with active recovery to decrease the effect of a Valsalva maneuver (Goldstein & Cheshire Jr., 2017). The HIIE bout consisted of repetitive 1‐min high intensity (70% estimatedWattmax) separated by 1‐min active recovery (10% estimatedWattmax) (Whitaker et al., 2022). After 10 min of HIIE, resistance was decreased to 15 W for the 2 min of cooldown. Five‐minute recordings commenced in a seated rest position at 5 and 30 min post‐HIIE.

## 3 AUTONOMIC RESPONSE AND VARIABILITY ASSESSMENT

The data was collected using an analog‐to‐digit unit (NI‐USB‐6212, National Instruments) and custom written MATLAB code (v2017a, The MathWorks, Inc., Natick, MA) using methods described previously (Boyne et al., 2023; Whitaker et al., 2022). Raw data were sampled at 500 Hz and then downsampled or resampled at 10 Hz. We extracted the data for beat‐to‐beat mean arterial pressure (MAP) (Finometer, Finapres Medical System, Amsterdam, The Netherlands) by obtaining mean area under each blood pressure waveform. The R waves of ECG QRS complex were determined using MATLAB code. Heart rate was calculated as beats per minute. We obtained power spectral density by using custom written MATAB code for BTB BPV and HRV. To examine the influence of the ANS on blood pressure, we measured BTB BPV in the temporal domain using beat‐to‐beat MAP CoV and in the spectral domain using LF PSD and HF PSD. PSD of BTB BPV obtained across the LF band (0.04–0.15 Hz) during rest is influenced by sympathetic nervous activity and vascular modulation. PSD of BTB BPV within the HF band (0.15–0.40 Hz) during rest is influenced by cardiac output, breathing, and or parasympathetic activity (Gianfranco Paratia et al., 2003; Parati et al., 2018; Stauss, 2007). During exercise BTB BPV LF PSD is influenced by sympathetic activity, metabolites, shear stress, and vasodilators, whereas HF band is influenced by cardiac output and breathing. To examine the influence of ANS function on heart rate, we measured HRV in the spectral domain using LF PSD and HF PSD. The PSD of HRV obtained in frequency domain of LF band (0.04–0.15 Hz) determines influence of sympathetic and vagal tone, whereas PSD HRV obtained in the HF band reflects modulation of vagal tone (Michael et al., 2017; Parati et al., 1995; Sandercock & Brodie, 2006). During exercising at higher intensities the HRV PSD within HF band reflects the influence of breathing (Michael et al., 2017; Sandercock & Brodie, 2006).

The PSD was determined using a Fast Fourier Transform (FFT) analysis applying the power spectral density (cPSD) software package in Matlab. We identified the periods of rest, high‐intensity bouts, cool down, and recovery periods as shown in Figure S1 for MAP and heart rate. Each time period signal was extracted, detrended via a quadratic function and concatenated to the following appropriate time period signals for high‐intensities and recovery bouts. For example, the first high‐intensity segments, and similarly the first recovery period to subsequent recovery periods. This created a stationary time series for computing the PSD. The PSD resolution was improved by interpolating the time series to obtain better resolution. The PSD was computed using a 100‐s Hanning window, and 50% superposition. Aligned with previous studies, the peak power of MAP and heart rate was determined across the power spectrum from 0.04 to 0.15 Hz for LF and 0.15 to 0.40 Hz for HF (Gianfranco Paratia et al., 2003; Parati et al., 1995). We then calculated the ratio of LF/HF to obtain sympathovagal balance using HRV. We calculated absolute power and area under the curve (AUC) for BTB BPV (mm^2^Hg/Hz) and HRV (beats/min^2^/Hz). For absolute BTB BPV we took the sum of PSD values within the LF and HF bands. AUC was calculated by the trapezoidal method similar to mentioned in other studies (Fonseca et al., 2022; Pruessner et al., 2003). We analyzed the area by the height and width of the LF and HF peaks across PSD. For the time domain analysis, we calculated BTB MAP CoV using the equation: (Young et al., 2020).

$$\mathrm{CoV}=\left(\text{Standard deviation}/\text{Mean}\right)\times 100$$

## 4 DATA ANALYSIS

Data analysis was performed using statistical package of social sciences (SPSS 27 version for Windows: SPSS Inc. Chicago, IL). A priori *α* was set as <0.05. We assessed the normality and sphericity using Shapiro Wilk test and Mauchly's test, respectively. Participant's characteristics were analyzed using one‐way ANOVA, and Fisher's exact test for categorical variables. Friedman's test was used for non‐normally distributed spectral measures of LF, HF, LF:HF ratio (AUC and absolute) and BTB BPV CoV. Post hoc comparisons used Wilcoxon's signed rank tests.

## 5 RESULTS

Twenty‐five datasets were available for our secondary analysis. We excluded 2 datasets due to noise in signal and therefore had a sample of 23 complete data sets for BPV, and for HRV. Since, this was a secondary analysis, an apriori sample size was not calculated. Other studies that reported blood pressure and heart rate variability during an acute bout of continuous incremental exercise reported a sample size of 15 participants which is smaller than the sample in this analysis (Cottin et al., 2008, 2010). The changes of an individual's PSD of MAP and heart rate across time point are shown in Figures S2 and S3.

### 5.1 Power spectral density (PSD)

All spectral measures of BTB BPV significantly changed across time, as shown in Table 2.

**TABLE 2:** Power spectral density of blood pressure variability and heart rate variability across timepoints.

*n* = 23	Baseline	High bout	Recovery bout	Cooldown	Post	Post 30	*p*‐value
HR (average)	76 ± 14	123 ± 15	113 ± 19	118 ± 19	92 ± 17	79 ± 12	<.001*
MAP (average)	74 ± 12	96 ± 13	89 ± 13	83 ± 12	74 ± 11	77 ± 13	<.001*
Low frequency for BPV mmHg^2^/Hz (0.04–0.15 Hz)							
Absolute PSD	876.42 ± 888.26	903.44 ± 438.66	1045.48 ± 464.03^a^	873.94 ± 762.62^c^	943.41 ± 700.08	1183.35 ± 971.42^a^	<.001*
AUC PSD	8.12 ± 8.76	8.17 ± 4.10	9.84 ± 4.41^ab^	8.09 ± 7.15^c^	8.97 ± 6.81	11.32 ± 9.59^ac^	<.001*
High frequency for BPV mmHg^2^/Hz (0.15–0.40 Hz)							
Absolute PSD	103.97 ± 77.44	356.44 ± 514.87^a^	354.90 ± 267.12^ab^	440.10 ± 362.47^ab^	275.41 ± 280.97^ac^	160.71 ± 153.47^abcde^	<.001*
AUC PSD	0.93 ± 0.68	3.43 ± 5.04^a^	3.41 ± 2.59^ab^	4.18 ± 3.47^ab^	2.67 ± 2.78^ac^	1.50 ± 1.46^abcde^	<.001*
Low frequency for HRV (beats/min)^2^/Hz (0.04–0.15 Hz)							
Absolute PSD	1470.01 ± 1571.01	224.18 ± 191.97^a^	625.96 ± 480.98^ab^	432.93 ± 443.23^abc^	1008.17 ± 736.59^bd^	1631.00 ± 1294.02^bcde^	<.001*
AUC PSD	13.76 ± 15.25	1.99 ± 1.75^a^	5.69 ± 4.40^ab^	3.99 ± 4.25^abc^	9.37 ± 6.99^bcd^	15.37 ± 12.52^bcde^	<.001*
High frequency for HRV (beats/min)^2^/Hz (0.15–0.40 Hz)							
Absolute PSD	632.84 ± 514.95	149.14 ± 315.96^a^	459.67 ± 427.30^b^	289.06 ± 397.05^abc^	436.75 ± 608.78^ab^	573.13 ± 590.96^bd^	<.001*
AUC PSD	5.83 ± 4.45	1.42 ± 3.02^a^	4.43 ± 4.17^b^	2.76 ± 3.84^abc^	4.19 ± 5.95^ab^	5.40 ± 5.53^bd^	<.001*
Low and high frequency ratio of HRV (LF/HF ratio)							
Absolute ratio	2.52 ± 1.83	4.06 ± 3.63	2.72 ± 3.00^b^	2.99 ± 2.67	3.36 ± 2.29^a^	3.90 ± 2.25^ac^	<.001*
AUC ratio	2.48 ± 1.82	3.80 ± 3.37	2.62 ± 2.96^b^	2.86 ± 2.34	3.29 ± 2.40	3.87 ± 2.29^ac^	<.001*

*Note*: Mean ± SD. Post‐hoc analysis was performed using Wilcoxon's Signed Rank test for power spectral density where (a) is significantly different from baseline; (b) is significantly different from high; (c) is significantly different from recovery; (d) is significantly different from cool‐down; (e) is significantly different from post.

Abbreviations: AUC, area under curve; Beats/min, heart rate per minute; BPV, blood pressure variability; HR, heart rate; Hz, Hertz; LF/HF, low frequency/high frequency; MAP, mean arterial pressure; mmHg^2^, millimeters per mercury square; PSD, power spectral density.

* Significantly different across time (*p* < .05) based on Friedman's statistical test.

#### 5.1.1 Blood pressure variability (BPV)

##### Low frequency (LF)

**FIGURE 1:**
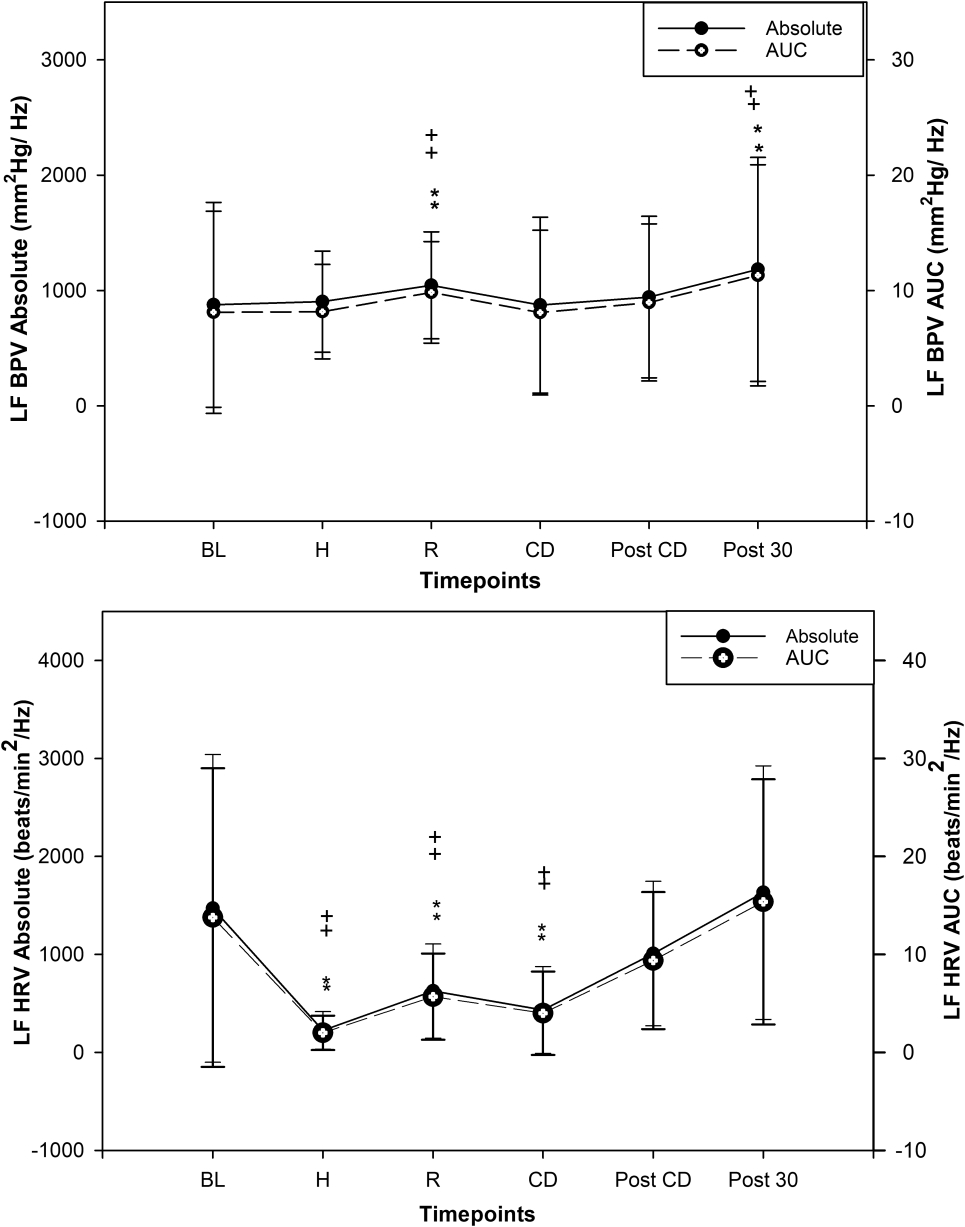
Beat‐to‐beat blood pressure variability (MAP) and heart rate variability, power spectral density (low frequency) response to an acute bout of high intensity interval exercise (HIIE, *n* = 23, mean ± SD); significantly different than baseline (rest) absolute power spectral density *(*p* < 0.05), **(*p* < 0.01). Significantly different than baseline (rest) area under curve of power spectral density ^+^(*p* < 0.05), ^++^(*p* < 0.01). MAP, mean arterial pressure; HRV, heart rate variability; LF PSD, low frequency power spectral density absolute values (closed dots); LF AUC, low frequency area under curve (open dots); Baseline, rest; High, low frequency during high intensity of HIIE; recovery, low frequency during active recoveries of HIIE; CD, active cool down; post CD, immediately post cool down; Post 30, post 30 min after HIIE; mm^2^Hg/Hz, millimeter square of mercury per hertz.

LF AUC PSD did not significantly increase during the high‐intensity bouts of HIIE compared to BL (*p* = 0.08). However LF AUC PSD was significantly higher during active recovery (*p* < 0.001) as shown in Figure 1. During high intensity, LF AUC PSD was lower than active recovery (*p* = 0.05). During cooldown and immediately post cool down, LF AUC PSD returned to BL and was not significant (*p* = 0.76, *p* = 0.28). Post 30‐min after HIIE, LF AUC PSD once again increased above BL (*p* = 0.002).

LF absolute PSD followed the same pattern as LF AUC PSD. LF absolute PSD did not significantly increase during the high intensity bouts of HIIE compared to BL (*p* = 0.08). However, LF absolute PSD significantly increased during active recovery (*p* = 0.04) as shown in Figure 1. During high intensity, LF absolute PSD was not significantly different than active recovery (*p* = 0.07). During cool down and immediately post cool down, LF absolute PSD returned to BL and was not significant (*p* = 0.80, *p* = 0.36). At 30 min after HIIE, LF absolute PSD increased from BL (*p* = 0.003).

##### High frequency (HF)

**FIGURE 2:**
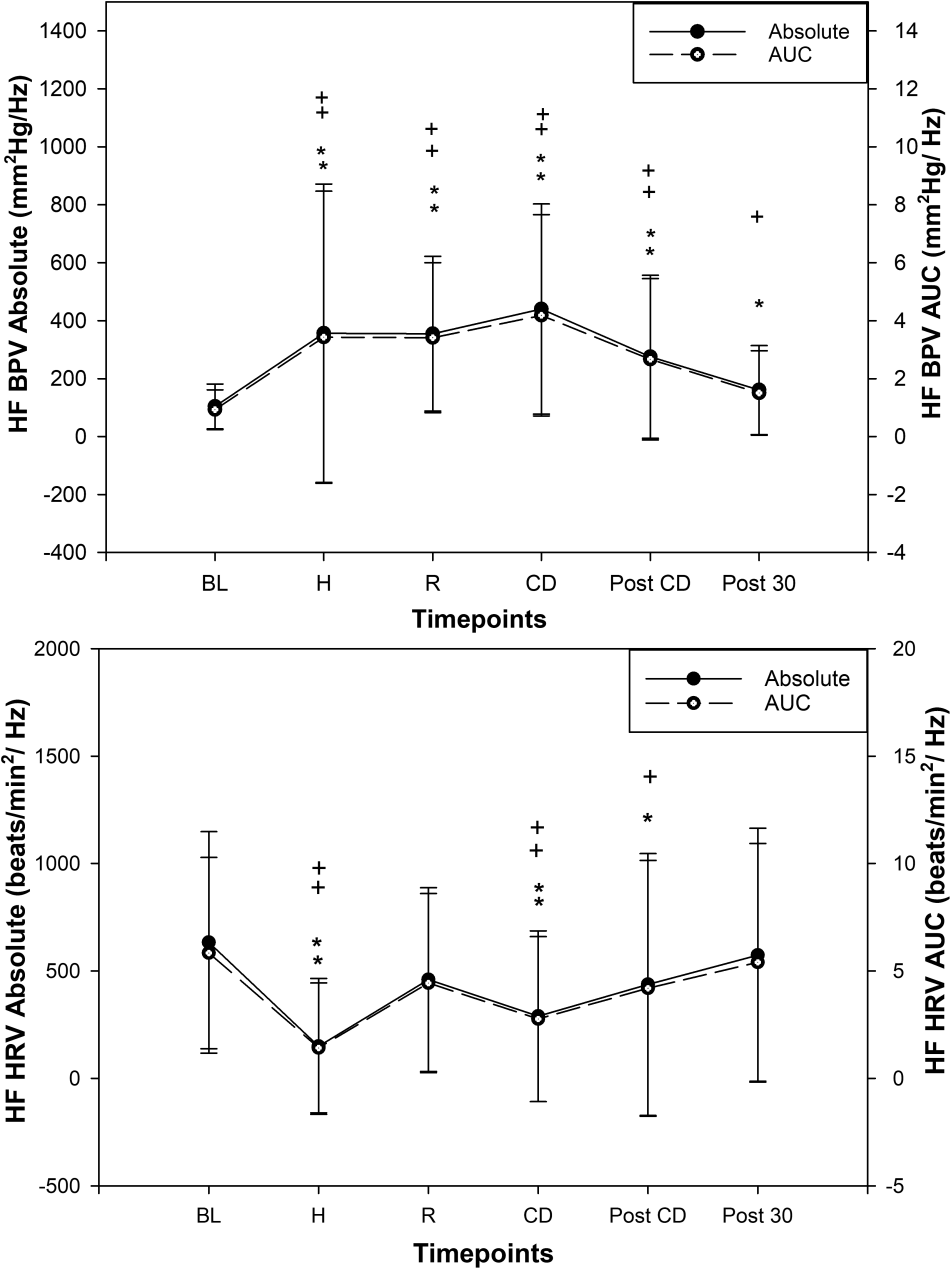
Beat‐to‐beat blood pressure variability (MAP) power spectral density (high frequency) response to an acute bout of high intensity interval exercise (HIIE, *n* = 23, mean ± SD); significantly different than baseline (rest) absolute power spectral density *(*p* < 0.05), **(*p* < 0.01). Significantly different than baseline (rest) area under curve of power spectral density ^+^(*p* < 0.05), ^++^(*p* < 0.01). MAP, mean arterial pressure; HRV, heart rate variability; HF PSD, high frequency power spectral density absolute values (closed dots); HF AUC; high frequency area under curve (open dots); Baseline, rest; High, high frequency during high intensity of HIIE; recovery, high frequency during active recoveries of HIIE; CD, active cool down; Post CD, immediately post cool down; Post 30, post 30 min after HIIE; mm^2^Hg/Hz, millimeter square of mercury per hertz.

HF AUC PSD significantly increased during the high‐intensity bouts of HIIE compared to BL (*p* < 0.01) and stayed higher during active recovery (*p* < 0.01) as shown in Figure 2. During high intensity, HF AUC PSD was lower compared to active recovery (*p* = 0.02). During the cooldown, immediately post cool down, and 30 min after HIIE, HF AUC PSD remained higher than BL (*p* < 0.01).

Once again, HF absolute PSD followed the same pattern as HF AUC PSD. HF absolute PSD was significantly increased during high‐intensity bouts of HIIE (*p* < 0.001) and stayed higher during active recovery bouts of HIIE (*p* < 0.001). During the high intensity, HF absolute PSD was lower compared to active recovery (*p* = 0.033). During the cooldown, immediately post cool down, and 30 min after HIIE HF absolute PSD stayed higher than BL (*p* < 0.001), (*p* < 0.001).

##### Co‐efficient of variation (CoV)

**FIGURE 3:**
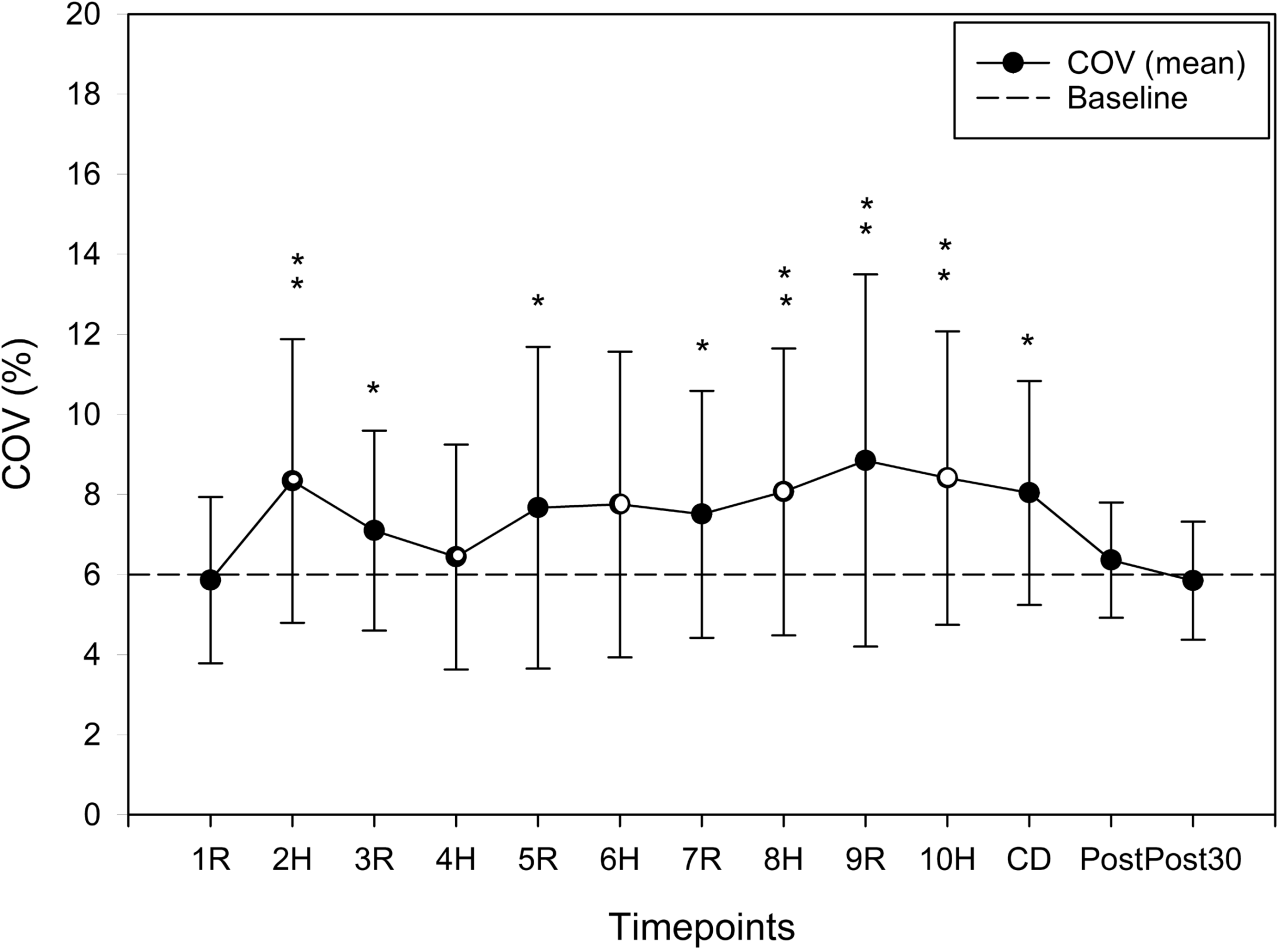
Coefficient of variation of beat‐to‐beat blood pressure variability (MAP) response to high intensity interval exercise (HIIE, *n* = 23, COV ± SD); Horizontal dashed line represents baseline value. Significantly different than baseline *(*p* < 0.05), **(*p* < 0.01). MAP, mean arterial pressure; COV, coefficient of variation; H, high intensity (open dots); R, active recovery (closed dots); CD, active cool down; Post, immediately post cool down; Post 30, post 30 min after HIIE.

There was significant change in BTB BPV CoV across time (*p* < 0.01). Compared to BL, BTB BPV CoV was increased during HIIE (*p* < 0.05), except active recovery bout minute 1, high‐intensity minute 4, and high‐intensity minute 6 were not significantly different than BL. While BTB BPV CoV increased during HIIE, it did not follow a consistent pattern when switching between high‐intensity and active recovery. Figure 3 shows the BTB BPV CoV returned to BL immediately following HIIE and 30 min after HIIE (*p* = 0.171 and *p* = 0.249 respectively).

#### 5.1.2 Heart rate variability (HRV)

##### Low frequency (LF)

By contrast to the blood pressure variability, LF AUC PSD for HRV significantly decreased during the high‐intensity bouts of HIIE compared to BL (*p* < 0.001) and stayed lower during active recovery (*p* = 0.004) as shown in Figure 1. During high intensity, LF AUC PSD was lower than active recovery (*p* < 0.001). During cooldown LF AUC PSD remained significantly lower than BL (*p* < 0.001), but returned to BL immediately post cool down (*p* = 0.23) and 30 min after HIIE (*p* = 0.40).

LF absolute PSD also significantly decreased during high intensity bouts of HIIE compared to BL (*p* < 0.001) and stayed lower during active recovery (*p* = 0.005). LF absolute PSD was also lower during high intensity compared to active recovery (*p* < 0.001). During cool down LF absolute PSD remained lower compared to BL (*p* < 0.001), and returned to BL immediately post cool down (*p* = 0.18) and post 30 min after HIIE (*p* = 0.45).

##### High frequency (HF)

HF AUC PSD for HRV significantly decreased during high‐intensity bouts of HIIE (*p* < 0.001). Interestingly, during active recovery bouts of HIIE, HF AUC returned to BL and was not significant (*p* = 0.17) as shown in Figure 2. During cool down and immediately post cool down, HF AUC PSD was significantly decreased compared to BL (*p* = 0.002 and *p* = 0.009). At 30 min after HIIE, HF AUC PSD returned to BL level (*p* = 0.20).

HF absolute PSD followed the similar pattern of HF AUC PSD. HF absolute PSD decreased significantly during high‐intensity bouts of HIIE (*p* < 0.001), but was not significantly different than BL during active recovery bouts (*p* = 0.14). During cool down and immediately post cool down, HF absolute PSD decreased significantly compared to BL (*p* = 0.002 and *p* = 0.007). At 30 min after HIIE HF absolute PSD returned to BL level (*p* = 0.20).

##### LF/HF ratio

**FIGURE 4:**
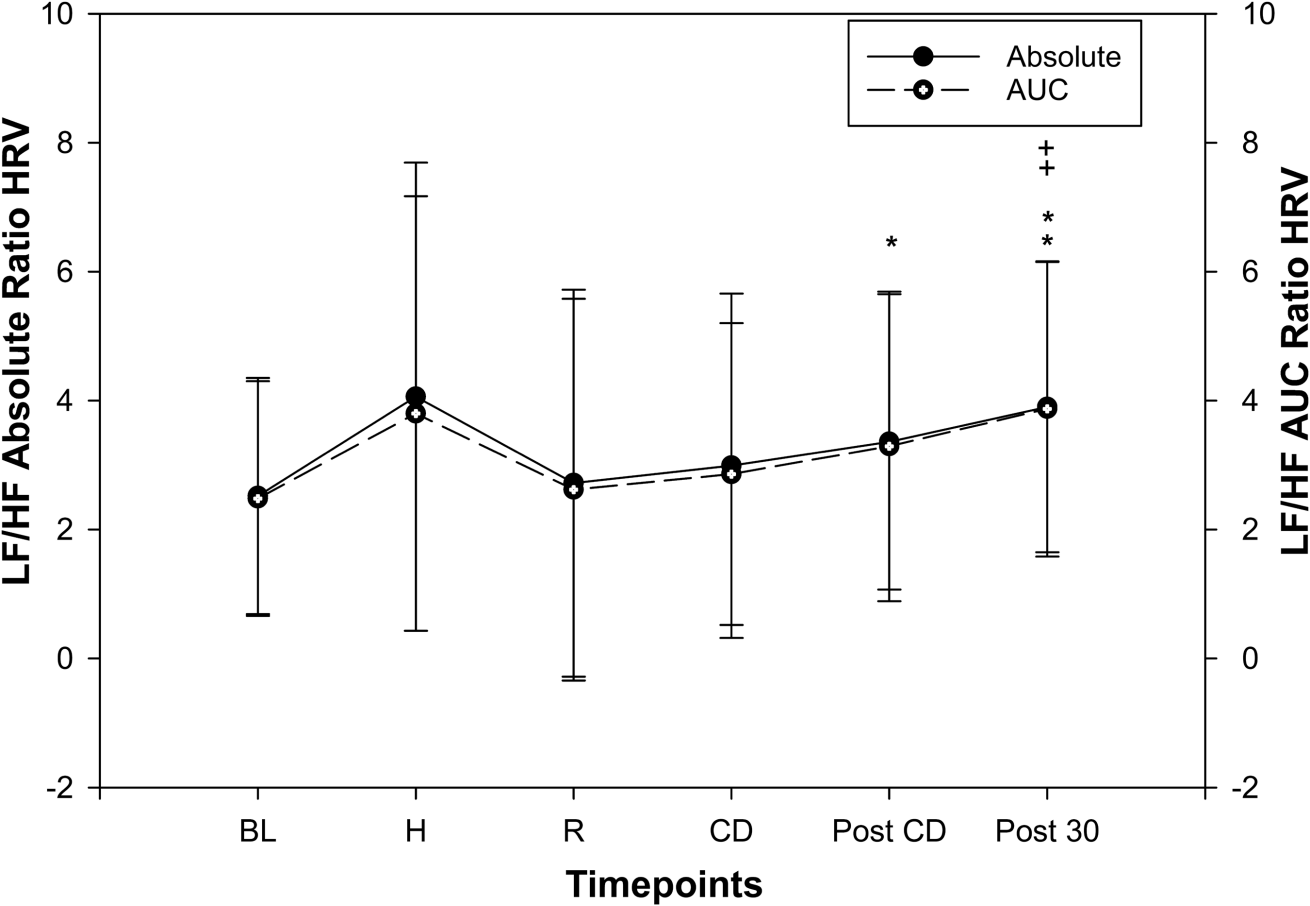
Heart rate variability (HRV) power spectral density (LF/HF ratio) response to an acute bout of high intensity interval exercise (HIIE, *n* = 23, mean ± SD); Significantly different than baseline (rest) absolute power spectral density *(*p* < 0.05), **(*p* < 0.01). ^+^Significantly different than baseline (rest) area under curve of power spectral density ^+^(*p* < 0.05), ^++^(*p* < 0.01); ratio PSD, low and high frequency ratio area under curve (open dots); baseline, rest; High, ratio of frequencies during high intensity of HIIE; Recovery, ratio of frequencies during active recoveries of HIIE; CD, active cool down; Post CD, immediately post cool down; Post 30, post 30 min after HIIE; mm^2^Hg/Hz, millimeter square.

The HRV LF/HF AUC ratio was not significantly different compared than BL during both high‐intensity and active recovery bouts of HIIE (*p* = 0.21 and *p* = 0.90), as shown in Figure 4. During cool down and immediately post cool down the LF/HF AUC ratio remained not significantly different than BL (*p* = 0.77 and *p* = 0.056). However, at 30 min after HIIE, the HRV LF/HF AUC ratio was significantly increased compared to BL (*p* = 0.004).

The HRV LF/HF absolute ratio during high‐intensity and active recovery bouts of HIIE also did not significantly change compared to BL (*p* = 0.15 and *p* = 0.93). During cool down, the LF/HF absolute ratio also remained at BL level. However, immediately post‐HIIE and 30 min after HIIE significantly increased compared to BL (*p* = 0.046 and *p* = 0.005).

## 6 DISCUSSION

While BPV and HRV have been characterized during continuous exercise, (Bartels et al., 2004; Cottin et al., 2008, 2010; Kaikkonen et al., 2012; Martinmäki et al., 2008) our study examines the acute effects of short‐interval, low‐volume HIIE on BTB BPV and HRV autonomic function. Performing repetitive switching in exercise intensity during HIIE in young adults, provides a unique challenge in BPV, HRV, and ANS modulation. Our main findings in support of our hypothesis includes: (1) BTB BPV significantly increased during HIIE in both LF and HF PSD compared to BL, (2) HRV during both the high intensity and active recovery bouts of HIIE was significantly lower than BL, and (3) interestingly, BTB BPV and HRV may differ during the active recovery bouts compared to the high intensity bouts of HIIE. In constrast to our hypothesis: (1) during recovery BTB BPV LF PSD returned to BL while HF PSD remained elevated. Similarly, HRV remained lower or returned to BL during recovery. A significant increase in BTB BPV CoV was seen across HIIE, with BTB BPV CoV returning to BL immediately post and 30 min after HIIE. HRV LF/HF ratio was not significantly different than BL during HIIE but was increased during recovery immediately post cool down and 30 min after HIIE. Our findings of increased BTB BPV and decreased HRV during HIIE are supported by previous studies performing continuous exercise (Cottin, 1999; Cottin et al., 2010; Perini et al., 2000; Sandercock & Brodie, 2006; Tulppo et al., 1996).

### 6.1 Blood pressure variability

#### 6.1.1 LF PSD during HIIE

Previous studies performing incremental low to high continuous exercise (Cottin et al., 2008) report concomitant increases in both LF and HF BTB BPV with increasing intensity. However, our results revealed LF PSD did not significantly increase during high intensity compared to BL. One potential reason for LF PSD not significantly increasing during the high intensity bouts could be due to the short 1 min bouts immediately interspered with active recovery. Therefore, limiting the amount of time for the BTB BPV to respond. Interestingly, during the active recovery bouts of HIIE, LF PSD was significantly increased compared to BL. One potential explanation for higher LF PSD during active recovery compared to high‐intensity could be due to a latency effect or delay in sympathetic activity during exercise by appoximately 30–60 s (Fisher et al., 2015; Seals et al., 1988; Seals & Victor, 1991). The duration of active recovery bouts could have been too short to allow for a decrease in sympathetic activity. Another potential mechanism that explains LF PSD increasing during active recovery is an increase sympathetic outflow via baroreceptors, which detects drops in blood pressure, due to the changes in the exercise intensities (Fadel & Raven, 2012; Fisher et al., 2015; Katayama & Saito, 2019). The ability of the peripheral vasculature to overcome the vasoconstriction response to supply blood flow to the exercising muscle, achieved by vasodilators released such as nitric oxide, adenosine, prostaglandins by vasculature, is known as “functional sympatholysis” and also may also explain no significant increase in BTB BPV LF PSD during high intensity (Cote et al., 2015; Fadel, 2008, 2015; Rowell, 1991).

#### 6.1.2 HF PSD during HIIE

HF PSD increased during high‐intensity and active recovery of HIIE compared to BL. HF PSD is a measure of the parasympathetic activity and respiration's influence on BTB BPV during rest. HF PSD during rest measures vagal parasympathetic activity which mediates fluctuations of heart rate on BP causing variability (Saul et al., 1991). During exercise parasympathetic activity withdraws and sympathetic activity increases which in turn increases heart rate and blood pressure (Fadel, 2015; Fu & Levine, 2013), however, it is important to note that various studies contribute HF PSD changes during exercise to changes in breathing and, ventilatory threshold (Cottin et al., 2008, 2010). Therefore, increased HF PSD during HIIE may be a reflection of increased breathing rather than parasympathetic activity and requires further exploration.

#### 6.1.3 LF and HF during recovery

Our findings during cool down and immediately post cool down determined LF PSD values returned to BL while HF PSD remained elevated. Previous studies have stated that during post exercise recovery the sympathetic activity withdraws, causing decreases in BP which may lead to post exercise hypotension. Our results show sympathetic activity (LF PSD) returned to BL during the HIIE cool down and immediately post cool down, however, it did not decrease below BL as hypothesized. Sympathetic activity increased 30 min after HIIE compared to BL, potentially to maintain tone of the blood vessel. HF PSD remained higher than BL immediately post cool down and 30 min after HIIE. Post‐exercise hyperapnea due to exercise oxygen debt leads to increased work of breathing and cardiac output, which may help explain the elevated HF PSD up to 30 min after HIIE (Coast & Krause, 1993; Elstad et al., 2018).

#### 6.1.4 Coefficient of variation during HIIE and recovery

BTB BPV CoV was also increased during HIIE. Interestingly, while MAP increased during high intensity and decreased during active recovery during HIIE (Whitaker et al., 2022), we did not observe a similar pattern in the BTB BPV CoV. This finding may be due to the influence of many different physiological variables on BTB BPV CoV during exercise, including neurotransmitters released by the autonomic nervous system at blood vessels (Fisher et al., 2015; Shoemaker et al., 1997), changes of intrathoracic pressure (Elstad et al., 2018; Forster et al., 2012), cardiovascular response based on the physical fitness, (Baker et al., 2020; O'Brien et al., 2021), and baroreceptor sensitivity (Fadel et al., 2001; Fisher et al., 2015). Immediately post cool down and 30 min after HIIE, BTB BPV CoV returned to BL, indicating a quick restoration of BP regulation following HIIE in young healthy adults.

### 6.2 Heart rate variability

#### 6.2.1 LF PSD during HIIE

Our findings of decreased HRV during HIIE are supported by previous studies reporting a linear decrease in HRV in response to incremental exercise up to high intensity. Various studies that have examined effects of continuous exercise on HRV have reported decreases in LF PSD (Fisher et al., 2009; Hautala et al., 2003; Martinmäki et al., 2008; Michael et al., 2017). Previous studies explain that decreases in LF PSD during exercise are observed due to a decrease in total peripheral resistance which shifts the spectral power to very low frequency waveband (Casadei et al., 1995; Perini et al., 1990; Sandercock & Brodie, 2006). One study observed HRV during an acute bout of HIIE and reported decrease in PSD during the high‐intensity bouts of HIIE (Edwards et al., 2022). Interestingly, our results determine that LF PSD is significantly higher during active recovery bouts of HIIE compared to the high intensity bouts. Therefore, low volume HIIE may be beneficial to cause autonomic modulation.

#### 6.2.2 HF PSD during HIIE

Our results showing a decrease in HF PSD during the high intensity bouts of HIIE are also supported by other studies performing continuous exercise. Other studies have shown consider HF PSD decrease occurs when intensity reaches at first ventilatory threshold or lactate threshold during incremental exercise (Cottin et al., 2006, 2007). The decrease in HF could be explained by non‐neural mechanisms such as effect of respiration on SA node, that shifts the spectral peak towards higher frequencies (Cottin et al., 2006; Perini et al., 2000). Therefore, potential explanation of decrease in HF PSD compared to BL during HIIE could be due to exercising at higher intensities above ventilatory threshold. Interestingly, active recovery bouts of HIIE allowed HF PSD to return to BL level. One potential explanation is that active recovery bouts allows oxygen consumption and recover from hyperpnea.

#### 6.2.3 LF and HF PSD during recovery

Our results showing a return of HRV back to BL values by 30 min after HIIE are consistent with previous studies. Following continuous exercises performed at 50% to 90% of maximal oxygen uptake showed LF and HF PSD recovery occurred with first 30 min to an hour after exercise (Burma et al., 2020; Perini et al., 1990; Sandercock & Brodie, 2006). One study reported effect of HIIE on HRV recovery across timepoints from immediately post low volume HIIE bout up to 8 hours and, did not find significant difference in spectral domain measures (Burma et al., 2020). Interestingly, we observed decreased LF and HF PSD immediately post an acute bout of low volume HIIE. The contrary results could be due to shorter duration of cool down around 2 min, inclusion of sedentary young individuals and greater sample size.

#### 6.2.4 LF/HF ratio

Various studies have used pharmacological and orthostatic stress changes on LF/HF ratio to understand sympathovagal balance (Pagani et al., 1986; van de Borne et al., 1997). Our results are contrary to other studies examining LF/HF ratio during continuous incremental exercise (Michael et al., 2017; Sandercock & Brodie, 2006). Studies that examined LF/HF ratio during incremental exercises showed that LF/HF ratio increases during low intensity exercise however decreases with increasing intensity (Hautala et al., 2003; Perini et al., 2000; Sandercock & Brodie, 2006). However, another study that examined an acute bout of HIIE, which consisted of 3 intervals of all‐out sprints, reported a decrease in LF/HF ratio at the end of exercise (Edwards et al., 2022). Therefore, interval exercise elicits different responses than continuous exercise and may be intensity dependent such as vigorous intensity compared to all‐out sprint intervals. Further work is needed to potentially test differing intensities and durations to understand BPV and HRV responses.

During recovery, our results showing an increase in HRV LF/HF ratio 30 min after HIIE is supported by another previous study reporting an increase within 5 min after HIIE. The observed increase in LF/HF ratio during recovery after an acute bout of HIIE was hypothesized to be due to sympathetic dominance (Burma et al., 2020). However, the HRV LF/HF ratio as a measure of sympathovagal balance during clinical experiments such as exercise has limited evidence and should be interpreted with caution (Goldberger, 1999).

## 7 LIMITATIONS

To gain reliable results of BTB BPV and HRV over 5 min, we detrended and concatenated each minute of high intensity and active recovery bout and used the data for analysis, future studies can consider longer HIIE bouts to obtain ample data for analysis specifically for examining alternating intensity of high and recovery bouts of HIIE effects on BTB BPV and HRV. Due to the nature of the secondary analysis, data was not available regarding baroreceptor sensitivity, which would provide information about baroreceptor role influencing blood pressure response during HIIE, this should be considered in future studies. Although, HRV and BTB BPV are a non‐invasive method to understand ANS function these metrices could also be affected by complex neural and non‐neural physiological mechanisms which should be considered in future research due to the descriptive nature of this secondary analysis.

## 8 FUTURE CONSIDERATIONS

Our results suggest that in young health individuals, HIIE as an exercise stimulus, increased ANS modulation and the cardiovascular system was able to rapidly respond examined by BTB BPV and HRV. Future studies should examine mechanisms such as cardiac output, vasomotor activity, baroreflex sensitivity, and breathing contributions to the changes in HRV and BTB BPV during HIIE (Cottin et al., 2010; Fadel, 2008; Lee et al., 2022; Raven et al., 2019). Autonomic dysfunction in various other populations such as diabetes and heart failure may also respond to HIIE differently and provide clinical insights about the cardiovascular and autonomic function interaction (Goldberger et al., 2019; Rafanelli et al., 2019; Solinsky et al., 2021). Our analysis performed short‐interval, low volume HIIE, however future studies could examine effects of short and long interval HIIE which would provide different stimulus to factors that alter the HRV and BTB BPV response (Crozier et al., 2018). This study lays the foundation for further studies of BPV and HRV in adults and exploring responses in adults with and without underlying cardiovascular disease or stroke, where these brain regions could be affected.

## 9 CONCLUSION

We show that HIIE increased autonomic modulation of blood pressure in terms of BTB BPV and heart rate in terms of HRV assessed by LF PSD, HF PSD, LF/HF ratio, and CoV. We also show BTB BPV and HRV was responsive to changes in intensity when switching between high intensity and active recovery bouts during HIIE in young healthy adults. BTB BPV and autonomic function returned to baseline values by 30 min after HIIE. Therefore, this study in young healthy individuals lays the foundation for characterizing a healthy BTB BPV and autonomic response to HIIE.

## AUTHOR CONTRIBUTIONS

S.W., A.A.W.H., S.A.B. conceived and designed research; A.A.W.H. performed experiments; S.W., M.C. analyzed data; S.W., A.A.W.H., S.A.B., M.C. interpreted results of experiments; S.W. prepared figures and tables; S.W., A.A.W.H., S.A.B. drafted manuscript; S.W., A.A.W.H., S.A.B., M.C. edited and revised manuscript.

## FUNDING INFORMATION

AAWH was supported by the National Heart, Lung and Blood Institute [T32HL134643], Cardiovascular Center's A.O. Smith Fellowship Scholars Program, Eunice Kennedy Shriver National Institute of Child Health and Human Development [T32HD057850], American Heart Association [898190], and Kansas Partners in Progress Inc. SAB was supported in part by the National Institute on Aging [P30 AG072973]. REDCap at University of Kansas Medical Center was supported by National Center for Research Resources [UL1TR000001]. The REACH laboratory was supported by Georgia Holland Endowment. The content is solely the responsibility of the authors and does not necessarily represent the official views of the NIH.

## CONFLICT OF INTEREST STATEMENT

None.

## ETHICS STATEMENT

This study was granted ethical approval by the University of Kansas Medical Center's Human Subjects Committee and Institutional Review Board. Prior to obtaining informed written consent, we provided verbal and written explanation of the experimental protocol, potential benefits and associated risks to all participants.

## Supporting information


Figure S1.

Figure S2.

Figure S3.

## Data Availability

Data will be made available upon reasonable request.
